# A genetically encoded anti-CRISPR protein constrains gene drive spread and prevents population suppression

**DOI:** 10.1038/s41467-021-24214-5

**Published:** 2021-06-25

**Authors:** Chrysanthi Taxiarchi, Andrea Beaghton, Nayomi Illansinhage Don, Kyros Kyrou, Matthew Gribble, Dammy Shittu, Scott P. Collins, Chase L. Beisel, Roberto Galizi, Andrea Crisanti

**Affiliations:** 1 Department of Life Sciences, Imperial College London, London, UK; 2 Department of Chemical and Biomolecular Engineering, North Carolina State University, Raleigh, NC USA; 3 Helmholtz Institute for RNA-based Infection Research (HIRI), Helmholtz-Centre for Infection Research (HZI), Würzburg, Germany; 4 Medical Faculty, University of Würzburg, Würzburg, Germany; 5 Centre for Applied Entomology and Parasitology, School of Life Sciences, Keele University, Keele, UK; 6 Department of Molecular Medicine, University of Padova, Padova, Italy

**Keywords:** Genetic engineering, Synthetic biology, Computational models, CRISPR-Cas9 genome editing

## Abstract

CRISPR-based gene drives offer promising means to reduce the burden of pests and vector-borne diseases. These techniques consist of releasing genetically modified organisms carrying CRISPR-Cas nucleases designed to bias their inheritance and rapidly propagate desired modifications. Gene drives can be intended to reduce reproductive capacity of harmful insects or spread anti-pathogen effectors through wild populations, even when these confer fitness disadvantages. Technologies capable of halting the spread of gene drives may prove highly valuable in controlling, counteracting, and even reverting their effect on individual organisms as well as entire populations. Here we show engineering and testing of a genetic approach, based on the germline expression of a phage-derived anti-CRISPR protein (AcrIIA4), able to inactivate CRISPR-based gene drives and restore their inheritance to Mendelian rates in the malaria vector *Anopheles gambiae*. Modeling predictions and cage testing show that a single release of male mosquitoes carrying the AcrIIA4 protein can block the spread of a highly effective suppressive gene drive preventing population collapse of caged malaria mosquitoes.

## Abstract (web summary)

Technologies that can halt the spread of gene drives would be highly useful in controlling or reverting their effect. Here the authors use the anti-CRISPR protein AcrIIA4 to inactivate drives in A. gambiae.

## Introduction

The management of vector and pest populations using nuclease-based gene drives is becoming a realistic possibility, particularly after the recent proof-of-principle demonstrations of genetic control technologies based on the broadly applicable CRISPR–Cas nucleases^1^. These technologies rely on the release of genetically engineered individuals that can rapidly propagate genetic constructs through wild populations together with the linked genetic modifications (e.g., knockout of sex determination^2^ or fertility genes^3^) or the introduction of genetic cargos (e.g., pathogen-killing molecules designed to block the development of parasites within the vector^4^). Several gene drive systems have been proposed and a few potential candidate strains have already been developed in the laboratory for the control of several organisms, including invasive rodents^5^, agricultural pests^6,7^ and disease vectors^2–4,8,9^. Access to effective ways to counteract the spread of gene drive elements is highly valued for risk mitigation and management alongside the implementation of these strategies: e.g., as a possible intervention in the case of unintended releases. This is particularly relevant for self-sustaining strategies showing high potential of spread, especially when these are set to control non-confined populations dispersed in large areas across multiple countries.

The first example of gene drive reversal systems was inspired by naturally occurring resistance to gene drives in the form of cleavage-refractory modification of the DNA sequence targeted by the driving endonuclease. Resistant alleles can pre-exist in the population as polymorphisms, or be generated de novo through non-homologous end-joining (NHEJ) repair of endonuclease-induced cleavage^10–13^. Anti-drive individuals could be genetically engineered to carry similar “drive-refractory alleles” and used to rescue the target population^8,10^. However, refractory alleles rely on a selective advantage conferred by the higher fitness compared to the drive, and therefore will have little effect on gene drives with low fitness cost (e.g., population-replacement drives)^14^. In addition, there are cases where tight functional constraints at the gene drive target sequence may hinder the development of this type of reversal approach, such as for the *dsx-*targeting gene drive that we recently developed in the malaria vector *Anopheles gambiae*^2^. Alternative reversal strategies involve the use of CRISPR components to cleave and replace DNA sequences specific to the gene drive construct with^15,16^ or without^17–19^ the use of an additional Cas9 expression cassette. Recently, guide RNA-only systems developed in *Drosophila* showed capacity to inactivate or replace gene drives in caged populations^19^. Although these strategies may offer the option to replace the drive with one or few “refractory alleles” or even restore the wild-type population, there are several complications attributable to the DNA-cleaving nature of the reversal that remain to be addressed, such as the formation and selection of resistant alleles and/or genomic rearrangement at the drive locus targeted by the reversal nuclease.

In this work, we describe the development and validation of a widely applicable genetic tool to counteract CRISPR-based gene drives by expressing a Cas9-inhibiting protein in a spatial and temporal manner coinciding with the gene drive activity, thereby circumventing problems related to additional cleavage of genomic sequences. We generated an *A. gambiae* transgenic line expressing the AcrIIA4 protein from the *Listeria monocytogenes* prophage^20–23^, and assessed its ability to both inhibit super-Mendelian inheritance of different driving constructs and prevent gene drive-mediated suppression of caged malaria mosquito populations.

## Results

### Generation of the vasa:A4 (anti-drive) line expressing the AcrIIA4 protein in the mosquito germline

**Fig. 1:**
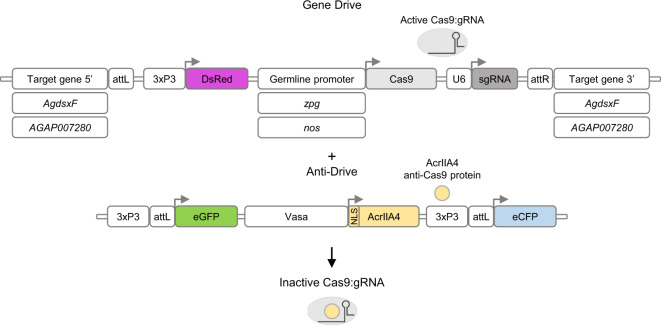
Schematic representation of gene drive and anti-drive constructs. Gene drive and anti-drive constructs respectively as inserted in the genome of  the gene drive lines previously generated (zpg:dsxF^2^; zpg:7280 and nos:7280, ref. ^26^) and the newly generated anti-drive (vasa:A4) line. Specifically, the gene drive constructs tested in this work are inserted at target sites within the *AGAP007280* or *AgdsxF* (*AGAP004050-RB*) gene-coding sequences and contain: the *Streptococcus pyogenes* Cas9 nuclease (Cas9), under the transcriptional control of the male and female germline-specific promoters *zpg* or *nos*; the gRNA, targeting the respective insertion site, transcribed by the RNA polymerase III responsive promoter (*U6*), and the DsRed fluorescent protein under the *3xP3* promoter (3xP3:DsRed) for the identification of larvae carrying the drive. The anti-drive construct carries the *Listeria monocytogenes* anti-CRISPR protein (AcrIIA4) expressed under the *vasa* male and female germline-specific promoter with the N-terminus addition of a nuclear localisation signal (NLS) and the eGFP fluorescent protein under the *3xP3* promoter (3xP3:eGFP) used for the screening of anti-drive positive larvae. The construct was inserted in a pre-existing docking line carrying the 3xP3:eCFP marker^25^. As a result the AcrIIA4 protein is expected to interact and inhibit the Cas9–gRNA complex, when co-expressed in the mosquito germline cells.

CRISPR-based homing gene drive constructs exploit the germline-specific action of the ribonucleoprotein complex (e.g., Cas9–gRNA), which is designed to recognise and cleave a DNA sequence homologous to the one where the drive-encoding construct is inserted. Double-strand breaks generated in premeiotic cells are preferentially repaired using the uncleaved homologous chromosome as a template through the homology-directed repair process, thus resulting in the drive-containing DNA strand being copied into the wild-type chromosome. As a consequence of germline maturation and subsequent fertilisation of a wild-type individual, the gene drive element is transmitted to the majority of the progeny, resembling a homozygous trait, and therefore increases its frequency in the population over generations. Aiming to inhibit this process, we genetically engineered an anti-drive transgenic line where the nuclear localisation signal (NLS)-tagged AcrIIA4 protein is expressed under the *vasa* promoter, transcriptionally active in the germline of both *A. gambiae* male and females^24^. As confirmed in vitro, fusion to various NLS tags does not perturb inhibition of Cas9 activity by AcrIIA4 (Supplementary Fig. 1). The anti-drive construct was inserted via embryo microinjection in a pre-existing docking line^25^. The identification of successful genomic integrations was based on the expected combination of fluorescent marker expression: the eGFP linked to anti-drive construct, and the eCFP already present at the docking site (Fig. 1). The progeny derived from a single eGFP (and eCFP) positive individual was used to establish the anti-drive transgenic line (identified as vasa:A4) after molecular confirmation of correct insertion of the construct (Supplementary Fig. 2a, b).

### Germline expression of AcrIIA4 imposes Mendelian transmission of gene drive constructs

**Fig. 2:**
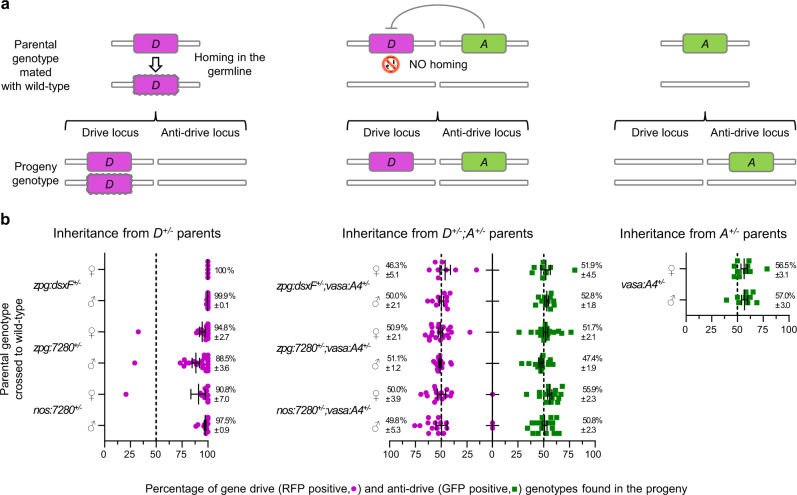
Germline expression of anti-CRISPR protein AcrIIA4 causes complete inhibition of gene drive homing. **a** Schematic representation of gene drive homing in the germline of individuals carrying one copy of the *drive* allele (*D*^*+/−*^): Cas9–gRNA-directed cleavage of the insertion site on the homologous wild-type chromosome is repaired via homology-directed repair (HDR), using the drive-carrying chromosome as template, resulting in the *D* allele being copied (the new *drive*copy is indicated as dashed magenta rectangle) and transmitted to most of the progeny (left). Illustration of gene drive homing inhibition in individual carrying one *drive* and one *anti-drive* copy (*D*^*+/−*^*;A*^*+/−*^) as consequence of AcrIIA4-directed Cas9–gRNA blockage, resulting in Mendelian inheritance of the *D* allele (middle). Mendelian inheritance of the *anti-drive* from *A*^*+/−*^ individuals (right). **b** Scatter plots showing the percentage of larvae carrying the gene drive (RFP positive) and/or the anti-drive (GFP positive) constructs from wild-type mosquitoes crossed to transgenic females or males carrying: only the gene drive construct, confirming high transmission rates (up to 100%) of the *D* allele from each of the transgenic lines tested (left); both gene drive and anti-drive constructs, showing Mendelian inheritance of both *D* and *A* alleles (middle); only the anti-drive construct, showing expected Mendelian rates of the *A* allele (right). Vertical dashed lines indicate the 50% Mendelian inheritance. Error bars indicate mean percentage values and standard error of the mean of transmission rates from all biological samples assessed for each cross. A minimum of seven biologically independent samples (ovipositing females) were examined over two independent experiments for each cross, with the exception of zpg:dsxF crosses, which were examined only once. The respective raw data are provided in the Source data file.

To test the AcrIIA4 inhibitory activity of gene drive homing in the mosquito germline, we crossed female mosquitoes hemizygous for the anti-drive construct (*vasa:A4*^*+/−*^) to the three most effective *A. gambiae* gene drive lines currently available. The zpg:dsxF line (previously identified as dsxF^CRISPRh2^) carries a driving construct, where the Cas9 from *Streptococcus pyogenes* (herein identified as Cas9) is expressed under the *zpg* regulatory elements, inserted within the sex-determining gene *AgdsxF* (*AGAP004050-RB*) to disrupt the production of the female splicing variant. Functional constraint of the targeted sequence allowed the driving construct to rapidly spread and eliminate caged mosquito populations by progressively reducing the reproductive capacity of females^2^. The zpg:7280 and nos:7280 lines (previously named zpg^CRISPRh^ and nos^CRISPRh^^26^) showed a dramatic improvement of the first generation of suppressive gene drives^3^ by tuning the temporal expression of the Cas9 through the use of the *zpg* and *nos* promoters. In both lines, homing of the CRISPR cassette disrupts the haplosufficient female fertility gene *AGAP007280*^26^. Male and female mosquitoes carrying one copy of both drive and anti-drive constructs (*zpg:dsxF*^*+/*−^*;vasa:A4*^*+/−*^, *zpg:7280*^*+/−*^*;vasa:A4*^*+/−*^ and *nos:7280*^*+/−*^*;vasa:A4*^*+/−*^) were crossed to wild-type individuals to assess fertility and analyse transmission frequencies of *drive* (*D*) and *anti-drive* (*A*) alleles to their progenies by screening for expression of the linked RFP (drive) and GFP (anti-drive) fluorescent markers. Individuals carrying only the drive or the anti-drive construct were also crossed to the wild-type counterpart as a control (Fig. 2a). Inheritance of the *anti-drive* allele from *vasa:A4*^*+/−*^ individuals was close to the expected Mendelian rates (average of 56.5% GFP positive from females and 57% from males), while all *drive*^*+/−*^ individuals transmitted the *drive* allele to most of the progeny as consequence of homing (averages ranging from 88.5% up to 100% of RFP positives found in the progeny). Notably, individuals carrying both drive and anti-drive transgenes showed a striking reduction of inheritance of the *drive* allele down to Mendelian rates (averaging from 46.3 to 51.1% of RFP positives), in line with complete blockage of Cas9–gRNA cleavage activity and consequent inhibition of germline homing (Fig. 2b).

### Fitness analysis of the vasa:A4 anti-drive line

Fertility assays did not show any substantial reduction in progeny outputs from individuals carrying one copy of the anti-drive construct (*D*^*+/−*^*;A*^*+/−*^ or *A*^*+/−*^) compared to the respective gene drive controls (*D*^*+/−*^), giving a minimum normalised value (NV) of larvae produced per females of 0.77 from *nos:7280*^*+/−*^*;vasa:A4*^*+/−*^ males (Welch’s *t* test *P* = 0.2544) and *zpg:7280*^*+/−*^*;vasa:A4*^*+/−*^ females (Welch’s *t* test *P* = 0.0732). On the other hand, a higher number of larvae (NV = 1.57) was obtained from *zpg:dsxF*^*+/−*^*;vasa:A4*^*+/−*^ females compared to *zpg:dsxF*^*+/−*^ females. Although this difference was not statistically significant when comparing independent oviposition counts from each cross (Welch’s *t* test *P* = 0.1035), the difference in overall number of larvae hatched was significant for all three crosses cited above (Fisher’s exact test). Notwithstanding that this type of phenotype assays may be affected by interindividual or batch variability, previous studies showed that parental deposition of Cas9 in the embryo can cause reduction of fertility in *D*^*+/−*^ females by generating embryonic mosaicisms for somatic null mutations at the targeted site, which are produced via NHEJ repairs^10,26^. These studies also indicated that the severity of this reduction varies among the different genes targeted and according to the promoter used to express the Cas9 protein, reflecting high levels of maternal depositions when the Cas9 expression is driven by the *vasa* promoter, compared to other regulatory sequences^10,26,27^. Consistent with these findings, maternally deposited AcrIIA4 from the *vasa* promoter may likely buffer somatic nuclease activity in *D*^*+/−*^*;A*^*+/−*^ embryos and therefore mitigate the impact of null mosaicisms on the fertility of the developed individuals, as evidenced by the increased *zpg:dsxF*^*+/−*^*;vasa:A4*^*+/−*^ female fertility compared to *zpg:dsxF*^*+/−*^ females (Supplementary Fig. 3a).

Further phenotypic characterisation of anti-drive individuals showed a moderate reduction in larval output (NV = 0.79, Welch’s *t* test *P* = 0.1398) from males carrying two copies of the anti-drive construct (*A*^*+/+*^) compared to the wild-type control tested in parallel. A similar reduction was also seen in *A*^*+/−*^ females (NV larvae per female = 0.79, Welch’s *t* test *P* = 0.0277); however, this was not reflected in *A*^*+/+*^ females that gave an average number of larvae comparable to wild-type (NV = 1.07, Welch’s *t* test *P* = 0.4168), indicating that this fertility impairments may not be directly caused by a double dose of AcrIIA4 in *A*^*+/+*^ individuals (Supplementary Fig. 3b, c). Furthermore, fertility assays from trans-hemizygous individuals, carrying one copy of the anti-drive construct and a marker construct inserted at the corresponding homologous locus (*vasa:A4*^*+*^*/mars*^*+*^) also showed similar reductions in larvae outputs compared to the wild-type control (NV = 0.67 and Welch’s *t* test *P* = 0.0147 from females, NV = 0.84 and Welch’s *t* test *P* = 0.1446 from males), indicating a moderate fitness cost likely associated with the locus of insertion (Supplementary Fig. 3c). The fraction of mated females recorded in these experiments also showed a possible impairment in mating competence that appeared more pronounced in *A*^*+/+*^ males, able to fertilise only 60% of the wild-type females tested compared to the 89% found in wild-type male crosses (Fisher’s exact test *P* = 0.0469). *A*^*+/−*^ males were instead able to fertilise 80% of the females (Fisher’s exact test *P* = 0.4924). Although not statistically significant, anti-drive females crossed to wild-type males showed a reduction in mating with 70% of *A*^*+/+*^ and 81% of *A*^*+/−*^ females able to mate in these settings (Supplementary Table 1).

### Single release of anti-drive males prevents elimination of caged mosquito populations by the suppressing CRISPR–Cas9 gene drive zpg:dsxF

**Fig. 3:**
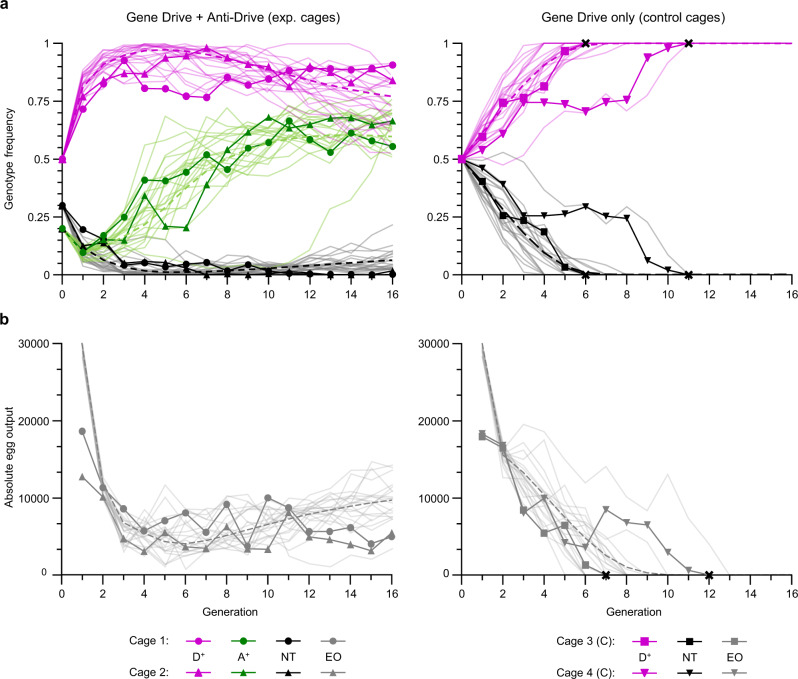
A single release of AcrIIA4 anti-drive males constrains gene drive spread preventing population suppression in caged mosquitoes. Two cages were initiated with a starting population of 600 *A. gambiae* mosquitoes of which 150 males and 150 females heterozygous for the *zpg:dsxF* driving allele (initial *drive* allele frequency of 25%), 120 homozygous-enriched males for the *vasa:A4* allele (initial *anti-drive* allele frequency of 20%) and 180 wild-type of which 30 males and 150 females to maintain equal sex ratio (left). In parallel, two control cages were established by releasing the same proportion of *drive* alleles (150 *zpg:dsxF*^*+/−*^ males and 150 *zpg:dsxF*^*+/−*^ females) and 300 wild-type mosquitoes (150 males and 150 females). **a** The frequency of RFP positive *drive* (D^+^, dark purple solid lines), GFP positive *anti-drive* (A^+^, dark green solid lines) and non-transgenic individuals (NT, black solid lines) was recorded for each generation by screening larvae for the expression of the respective fluorescent markers. **b** Absolute number of eggs produced each generation (dark grey solid lines). Genotype frequencies (D^+^, A^+^ and NT) and egg output (EO) values were overlapped to the respective deterministic (dotted lines) and stochastic (light-coloured lines) model simulation based on the parameters provided in Supplementary Table 2. The respective raw data are provided in the Source data file.

Fitness values and transmission rates of the respective alleles were used to generate deterministic and stochastic models to simulate drive and anti-drive genotype frequencies (over discrete generations) after a single release of vasa:A4 males into a defined population, where individuals carrying the zpg:dsxF gene drive (previously developed by Kyrou et al.^2^) were introduced. We tested our model predictions by releasing 20% of vasa:A4 males, after fluorescence microscope-based enrichment for homozygotes (Supplementary Fig. 2c, d), into a population of 600 mosquitoes in total, including 50% heterozygous zpg:dsxF (males and females) and 30% wild-type individuals. Two experimental replicates were performed in parallel with two control cages, where 300 heterozygotes for the zpg:dsxF drive were added to 300 wild-type individuals. Inheritance of drive and/or anti-drive constructs and total egg output was measured at each generation from the four cages. As expected, the frequency of zpg:dsxF genotypes increased rapidly in all cages, leading to elimination of both control populations after the 6th and 11th generation, as a consequence of the progressive reduction of egg output. Consistent with model predictions, after an initial increase, the zpg:dsxF spread was halted in the cages where vasa:A4 males were released, maintaining frequencies between 98.1 and 76.6% after generation 3, and an average of RFP positive individuals of 87.3% ± 0.05 SD from G3 to G16. Despite not having an inbuilt homing machinery like the zpg:dsxF drive, the frequency of vasa:A4 carrying individuals also increased gradually from the ~10% at G1 up to a maximum of 68.1% (Fig. 3a). As also shown by the model simulation, the gradual increment of anti-drive frequency is determined by the higher overall fitness linked to the *vasa:A4* alleles compared to the *drive* (Supplementary Table 2). On the other hand, the egg output of the two experimental cages was maintained above one-fifth of the generation after release, with values (relative to G1) of 0.27 and 0.43 at generation 16, when the last measurement was performed (Fig. 3b). To determine the contribution of nuclease-resistant alleles over generations, we performed amplicon sequencing of pooled samples collected at generations 1, 5, 10 (from experimental and control cages) and 15 (for experimental cages). In line with modelling prediction, a progressive accumulation of mutations at the *dsx*-target site was observed in the control cages, reaching a total frequency of 0.047 and 0.062 (among non-drive alleles) in the generations prior to population collapse. On the contrary, mutations measured from the cages containing vasa:A4 carrying mosquitoes remained at low levels and gradually decreased reaching minimum total frequency at G15 (0.013 and 0.019; Supplementary Fig. 4a). This result is consistent with no significant end-joining resistance being generated from mosquitoes carrying both the drive and anti-drive constructs (Supplementary Fig. 4b). Although not accounting for spatial dispersal, our models show a long-term equilibrium between *drive*, *anti-drive* and *wild-type* alleles (Supplementary Fig. 5) that is primarily, although not exclusively, dependent on their relative fitness (Supplementary Fig. 6), as described previously for other reversal strategies^28–30^. Allele dynamics over an extended number of generations shows that a single release of vasa:A4 anti-drive males can sensibly constrain the gene drive spread, preventing it from reaching high frequencies and drastic reproductive load according to the drive/anti-drive relative frequency at release, even when the fitness of females heterozygous for the drive is considered equal to wild-type, thereby facilitating the spread of the drive (Supplementary Fig. 6).

## Discussion

In this work, we demonstrate that a genetically encoded anti-CRISPR protein can be highly effective in inhibiting potent CRISPR-based gene drives engineered to suppress malaria mosquitoes by restoring their inheritance to Mendelian rates. Our experiments show that a single low frequency release of males carrying the anti-CRISPR construct is highly effective in preventing population suppression, despite a reduced fitness associated with the anti-drive.

We successfully tested wide applicability of the AcrIIA4-based anti-drive technology against different gene drive systems, previously engineered for population suppression, and achieved proof-of-concept of propulation rescue in caged insects. Here, gene drive inhibition is achieved via protein–protein interaction, rather than DNA cleavage of drive-specific sequences, bypassing potential unintended effects of genome editing alterations caused by alternative Cas9–gRNA or gRNA-only reversal strategies^15,17–19^. Repair of CRISPR-mediated cleavage of genetic components encoding for the gene drive may alter either the activity or the specificity of action with unpredictable consequence in the long term; for example, upon modification of the targeting sequence of the driving gRNA. Moreover, expression of anti-CRISPR proteins can be easily restricted to the cell types where the gene drive is active by using tissue-specific pol II promoters (e.g., the *vasa* promoter used here), while gRNA-based methods generally require ubiquitously active pol III promoters. The broad spectrum of DNA mimic proteins^31,32^ expands dramatically the application range of this system, which in principle could be used to counteract any genetic control technology employing CRISPR–Cas9 tools, without requiring ad hoc engineering for different genetic constructs. This aspect may be particularly advantageous in the case of synonymous or silent mutations arising at the driving genetic sequence (naturally or induced by DNA-cleaving reversal construct) that may prevent the effect of counteracting systems involving DNA cleavage. In addition, the generation and selection of resistance to the anti-drive is reduced compared to DNA-cleaving methods, for which creation of resistant alleles at the drive target is expected at each generation as shown for homing gene drive constructs^10–13^. It should also be considered that risks related to remobilisation of the anti-drive protein inhibitor are theoretically reduced compared to other strategies. For instance, in the case a gene drive-targeting gRNA^17^ may become genetically linked to a mutated Cas9 sequence that remains functionally active, but resistant to further cleavage by the reversal gRNA^19^. Another aspect to be considered is that broadly active anti-CRISPR proteins, conferring low fitness costs to the carriers, have the potential to persist in a population over time, effectively generating a genetic barrier against newly introduced gene drives or self-limiting genetic control strategies, which rely on CRISPR–Cas with overlapping spatiotemporal activity.

As for gene drives, anti-drive technologies such as the one developed here will also require careful experimental and mathematical evaluations before these can be considered as an effective and field-applicable countermeasure. The effectiveness of this technology in blocking gene drive spread will ultimately depend on the relative fitness of drive and anti-drive constructs, as well as rates and fitness cost associated with genetic resistance against the drive or anti-drive, although the latter is expected to be reduced for our protein-based inhibitor compared to DNA-cleaving approaches. The anti-CRISPR tool described here will also be of great use to expedite laboratory husbandry of CRISPR–Cas expressing transgenic lines, including suppressive gene drives, which usually require continuous backcrossing to wild-type strains. Finally, this work provides an additional technology for genetic regulation of CRISPR activity that can be of great use for a broad range of applications in *A. gambiae* mosquitoes, as well as other medically relevant insects, including the model insect *Drosophila melanogaster*.

## Methods

### Anti-CRISPR testing in cell-free reactions

*Escherichia coli* cell-free reaction mixture was sourced from Arbor biosciences (Arbor Biosciences, Cat: 507024). Each 75 µL, 1.25×-concentrated MyTXTL reaction was loaded with the necessary DNA expression templates and ultimately divided into 5-µL individual reaction droplets for incubation, expression and fluorescence monitoring, as described in ref. ^31^ (Supplementary Fig. 1a). To prevent the degradation of linear DNA templates, GamS (Arbor Biosciences, Cat: 501024) was added to the 75 µL TXTL reaction master mix at a final concentration of 2 µM (ref. ^33^). The anti-CRISPR protein AcrIIa4 (SPC gb013) and Cas9 sgRNAs were expressed from linear template DNA at 1 and 4 nM concentrations, respectively. Cas9 (pCB843) and deGFP (pCB556) were expressed from plasmid DNA templates at 1 and 0.5 nM concentrations, respectively. The reactions were mixed by brief vortexing and collected using a benchtop centrifuge. Each reaction was split into two aliquots, each of 5 µL, and loaded into a 96-well V-bottom plate (Corning Costar 3357) and covered with a cap mat. The 96-well plate with TXTL droplets was loaded into a BioTek Synergy H1 plate reader at 29 °C without shaking. Fluorescence of TXTL reaction was measured at Exc. 485 nm, Em. 528 nm every 3 min, for 16 h. Only the fluorescence from the endpoint of the reaction was reported (Supplementary Fig. 1b).

### Plasmid construction

The *L. monocytogenes* AcrIIA4-coding sequence, synthetised and codon-optimised for *A. gambiae* (ATUM), was amplified using primers containing the XhoI cleavage site followed by a NLS at the N-terminus side and the PacI site after the C-terminus (RG247–RG248; Supplementary Table 3). The fragment was digested and ligated into a pre-existing vector containing the *vasa* promoter and terminator sequences^24^ flanking the XhoI and PacI sites, the eGFP-coding sequence under the control of the 3xP3 promoter separated by the ϕC31 attB recombination sequence, resulting in the final plasmid named C77 (Genbank accession code MZ172909**)**.

### Microinjection of embryos and selection of transformed mosquitoes

All mosquitoes used in this work were reared under standard conditions of 80% relative humidity and 28 °C. Adult mosquitoes of a previously generated *A. gambiae* attP docking line^25^ were blood-fed by Hemotek and freshly laid embryos were aligned for microinjections, as described previously^34^. The injected solution contained 50 ng/μL of the vasa:AcrIIA4 construct and 400 ng/μL of a helper plasmid expressing the φC31 integrase under the *vasa* promoter^3^. Hatched larvae were screened for transient expression of the eGFP marker and crossed to wild-type mosquitoes to obtain transgenic individuals expressing both eGFP and eCFP. Expression of fluorescent markers was analysed on a Nikon inverted microscope (Eclipse TE200).

### Molecular confirmation of insertion and zygosity assessment

Vasa:A4 and wild-type mosquitoes were used for genomic DNA extraction, using Qiagen blood and tissue kit (Qiagen) followed by PCR amplifications at the insertion locus to confirm the correct integration of the transgene and zygosity of the vasa:A4 released in the cage trial. The ϕC31-mediated integration of the vasa:A4 construct was confirmed using primers binding the integrated construct and the neighbouring genomic locus, using the RG1044 and RG187 primers (PCR A; Supplementary Fig. 2a, b). The proportion of heterozygous (*vasa:A4*^*+/−*^) and homozygous (*vasa:A4*^*+/+*^) anti-drive males released in the cage trial was determined, using the RG1044-5R1 primers binding the transgene and the flanking genomic region (PCR B) and primers RG1047–RG1044 binding either side of the transgene insertion site (PCR C). A total of 37 male individuals were assessed for cage 1 and 25 male individuals for cage 2 (Supplementary Fig. 2a, c, d and Supplementary Table 3).

### Mosquito genetic crosses

Vasa:A4 males carrying one copy of the anti-drive construct (*vasa:A4*^*+/−*^) were crossed to heterozygous females of each gene drive line (*zpg:dsxF*^*+/−*^, *zpg:7280*^*+/−*^ or *nos:7280*^*+/−*^). Larvae carrying one copy of the *drive* (RFP positive), one copy of the *anti-drive* (GFP positive) or both (RFP and GFP positive) were selected and crossed to wild-type individuals for phenotypic assays (Supplementary Fig. 3a).

Vasa:A4 males were crossed to virgin females carrying a 3xP3:DsRed marker in the same locus (mars transgenic line^25^) to generate individuals carrying either both transgenes (*vasa:A4*^*+*^*/mars*^*+*^), and therefore homozygous for the disruption of the genetic locus (GFP and RFP positive) or either transgene in heterozygosity (GFP positive *vasa:A4*^*+/−*^ and RFP positive *mars*^*+/−*^). For each genotype, transgenic males and females were crossed to wild-type individuals for phenotypic characterisation (Supplementary Fig. 3b). Transgenic individuals carrying both transgenes (*vasa:A4*^*+*^*/mars*^*+*^) were also crossed to each other to generate individuals homozygous either for the vasa:A4 (*vasa:A4*^*+/+*^) or the mars (*mars*^*+/+*^) construct, as well as siblings carrying one copy of each construct (*vasa:A4*^*+*^*/mars*^*+*^). Males and females of each genotype were crossed to wild-type for phenotypic characterisation (Supplementary Fig. 3c).

### Phenotypic assays

For each genotype tested, 30 transgenic male or female adults were crossed to an equal number of wild-type mosquitoes for 5 days, blood-fed and a minimum of 15 females allowed to lay individually. The entire egg and larval progeny were counted for each lay (Supplementary Fig. 3). Females that failed to give progeny and had no evidence of sperm in their spermathecae were excluded from fertility analysis, but considered for mating analysis (Supplementary Table 1). To confirm parental zygosity of the vasa:A4 alleles, progenies were also screened for the presence of WT individuals (negative to fluorescence screening).

Inheritance of gene drive (RFP positive) and anti-drive (GFP positive) transgenes was measured by screening the entire larval progeny obtained from each oviposition. Females that produced less than ten larvae were excluded from the analysis of transgenic inheritance rates (Fig. 2b).

Statistical differences against selected reference crosses tested in parallel were assessed using Welch’s unpaired *t* test, for both larval and egg output averages, and Fisher’s exact test for the total number of larvae hatched from each cross (Supplementary Fig. 3).

### Discrete-generation (non-overlapping) cage trial

To minimise possible parental bias of Cas9–gRNA deposition and consequent generation of alleles resistant to the drive, the gene drive individuals released in the cage trial were obtained from both zpg:dsxF males crossed to wild-type females and zpg:dsxF females crossed to wild-type males in equal numbers. These were subsequently mixed at L1 stage and reared in parallel with the offspring of *vasa:A4*^*+/−*^ males crossed to *vasa:A4*^*+/−*^ females, and wild-type insects. RFP positive gene drive and GFP positive anti-drive larvae were screened at L3 stage, and the developing male and female pupae were sexed and allowed to emerge in individual cages in parallel with wild-type males and females. *Vasa:A4*^*+/+*^ individuals used for the release were selected based on higher intensity of the eGFP signal from larval progeny of vasa:A4 heterozygous parents. Adult mosquitoes were mixed only when all the pupae had emerged.

Two experimental cages were initiated by releasing 150 *zpg:dsxF*^*+/−*^ males and 150 *zpg:dsxF*^*+/−*^ females (corresponding to a 25% allele frequency at the gene drive locus) together with 120 anti-drive males enriched for homozygous (~20% allele frequency of anti-drive alleles), 30 wild-type males and 150 wild-type females (contributing 30% to the total of ~80% allele frequency of wild-type alleles at the anti-drive locus and 75% for the drive locus). In parallel, two control cages were initiated by releasing an equal number of gene drive mosquitoes (150 *zpg:dsxF*^*+/−*^ males and 150 *zpg:dsxF*^*+/−*^ females) with 150 wild-type males and 150 wild-type females (corresponding to 25% allele frequency of the gene drive).

Each generation, mosquitoes were left to mate for 5 days before they were blood-fed on anaesthetised mice. Two days later, egg bowls filled with water and lined with filter paper were added in the cages to allow for overnight oviposition. The following day, eggs laid in the egg bowl were dispersed using gentle water spraying to homogenise the population, and 650 eggs were randomly selected to seed the next generation. The remaining eggs were photographed and counted using JMicroVision V1.27 to obtain the overall egg output from each cage (Fig. 3b and Source data file). Larvae hatching from the 650 eggs were counted and reared at a density of 200 per tray (in ∼0.5 litre of water). L2/L3 larvae were screened for the presence of the RFP and GFP marker to measure gene drive and anti-drive genotype frequencies (Fig. 3a and Source data file). All the pupae obtained from the 650 eggs were used to seed the following generation.

### Animal husbandry

Mosquitoes in the cage trials were fed using anaesthetised 2–6 month old female mice (CD1 strain). CD1 mice were kept in social-housing caged units with 12/12 light–dark cycles, 45–65% relative humidity and at temperature of 20–24 °C. All animal work was conducted according to UK Home Office Regulations and approved under Home Office License PPL 70/8914 by the AWERB at Imperial College London.

### Amplicon sequencing analysis

Adult mosquitoes were collected at G1, G5, G10 and G15 from each of the four cages after obtaining the respective progenies (Fig. 3). DNA extraction from pooled individuals, PCR amplification and amplicon sequencing were performed for each of the 14 samples as previously described^2^ using the 4050-Illumina-F and 4050-Illumina-R primers. Raw amplicon sequencing data were deposited in the EBI-ENA database (accession code PRJEB44729). The CRISPResso v1.0.8 software^35^ was used to analyse the frequency of wild-type and mutated sequences at the *zpg:dsxF* gene drive target as previously described^2^, accounting for all indels and substitutions present at the target sequence  and/or any of the two invariable nucleotides of the  corresponding PAM sequence (-GG). Exogenous contaminant alleles were removed bioinformatically^2^ (Supplementary Fig. 4a).

### Modelling

Discrete-generation recursion equations were used for genotype frequencies, with males and females treated separately as in refs. ^2,11,25^. Here, we model two loci: the gene drive locus, where we consider three alleles, *W* (wild-type), *D* (drive) and *R* (non-functional nuclease-resistant), and the anti-drive site with two alleles *W* (wild-type) and *A* (anti-drive). ${F}_{{ij|kl}}(t)$ and ${M}_{{ij|kl}}\left(t\right)$ denote the genotype frequency of females (or males) in the total population, where the first set of indices denotes alleles at the target locus ${ij}={\{WW},{WD},{WR},{DD},{DR},{RR\}}$, and the second set denotes the anti-drive locus, ${kl}={\{WW},{WA},{AA\}}$. For simplicity, we assume full recombination and no linkage between the loci. There are 18 female genotypes and 18 male genotypes (see list in Supplementary Table 2); 6 types of eggs in proportions ${E}_{{W|W}},{E}_{{W|A}},{E}_{{D|W}}$,$\,{E}_{{D|A}}$, ${E}_{{R|W}}$, ${E}_{{R|A}}$, where the first index refers to the target site allele and the second to the anti-drive; and similarly 6 types of sperm, ${S}_{{W|W}},{S}_{{W|A}},{S}_{{D|W}},{S}_{{D|A}}$, ${S}_{{R|W}}$, ${S}_{{R|A}}$.

Homing of the gene drive is assumed to occur only when the anti-drive is not present. Adults of genotype ${WD|WW}$ (i.e., with no anti-drive) produce gametes at meiosis in the ratio ${W|W:D|W:R|W}$ as follows: $(1-{d}_{f})(1-{u}_{f}):{d}_{f}:(1-{d}_{f}){u}_{f}$ in females, $\left(1-{d}_{m}\right)\left(1-{u}_{m}\right):{d}_{m}:\left(1-{d}_{m}\right){u}_{m}$ in males. Here, ${d}_{f}$ and ${d}_{m}$ are the rates of transmission of the driver allele in the two sexes, and ${u}_{f}$ and ${u}_{m}\,$ are the fractions of non-drive gametes at the target site that are repaired by meiotic end-joining and are non-functional and resistant to the drive (*R*). If the anti-drive is present (*WD*|*WA* and *WD*|*AA*), drive inheritance is Mendelian. In all other genotypes, inheritance at the target site is also Mendelian. In the deterministic model, fitness effects are manifested as differences in the relative ability of female or male genotypes to participate in mating and reproduction. We let ${w}_{{ij|kl}}\le 1$ represent the fitness of genotype ${ij|kl}$ relative to ${w}_{{WW|WW}}=1$ for the wild-type homozygote (see “overall fitness” in Supplementary Table 2). We assume the *dsx*-target gene is needed for female fertility, thus females with *DD*, *DR* and *RR* at the gene drive locus are sterile.

We firstly consider the gamete contributions from each genotype. The proportions ${E}_{{m|n}}(t)$ with allele $m={\{W},D,{R\}}$ at the gene drive locus, and $n={\{W},{A\}}$ at the anti-drive locus in eggs produced by females participating in reproduction are given in terms of the female genotype frequencies ${F}_{{ij|kl}}\left(t\right)$:

$${E}_{m{{\rm{|}}}n}\left(t\right)=\frac{{\sum }_{i=1}^{3}{\sum }_{j=i}^{3}{\sum }_{k=1}^{2}{\sum }_{l=k}^{2}{{c}_{{ij}{{\rm{|}}}{kl}}^{m,n}{w}_{{ij}{{\rm{|}}}{kl}}F}_{{ij}{{\rm{|}}}{kl}}\left(t\right)}{{\sum }_{i=1}^{3}{\sum }_{j=i}^{3}{\sum }_{k=1}^{2}{\sum }_{l=k}^{2}{{w}_{{ij}{{\rm{|}}}{kl}}F}_{{ij}{{\rm{|}}}{kl}}\left(t\right)}$$

where *i* and *j* are each summed such that {1, 2, 3} corresponds to {*W*, *D*, *R*} and *k* and *l* such that {1, 2} corresponds to {*W*, *A*}. The coefficients ${c}_{{ij|kl}}^{m,n}\,$ correspond to the proportion of the gametes from female individuals of type (${ij|kl}$) that carry alleles (${m|n}$). For example, assuming no linkage, for a female of genotype *WD*|*WA*, the coefficient for alleles of type ${m|n}={W|W},{W|A},{D|W\; and\; D|A}$ is =¼, since inheritance of the drive is Mendelian due to the presence of anti-drive in that genotype, and is zero for alleles of type ${R|W}$ and ${R|A}$, since it is assumed that no end-joining resistance is generated with anti-drive present. An analogous expression is used for sperm:

$${S}_{m{{\rm{|}}}n}\left(t\right)=\frac{{\sum }_{i=1}^{3}{\sum }_{j=i}^{3}{\sum }_{k=1}^{2}{\sum }_{l=k}^{2}{c}_{{ij}{{\rm{|}}}{kl}}^{m,n}{{w}_{{ij}{{\rm{|}}}{kl}}M}_{{ij}{{\rm{|}}}{kl}}\left(t\right)}{{\sum }_{i=1}^{3}{\sum }_{j=i}^{3}{\sum }_{k=1}^{2}{\sum }_{l=k}^{2}{w}_{{ij}{{\rm{|}}}{kl}}{M}_{{ij}{{\rm{|}}}{kl}}\left(t\right)}$$

To model cage experiments, the initial frequency of heterozygote drive females and males is ${F}_{{WD|WW}}={M}_{{WD|WW}}=0.25$, of anti-drive males ${M}_{{WW|AA}}=0.2$, and of wild-type female and males ${F}_{{WW|WW}}=0.25$ and $\,{M}_{{WW},{WW}}=0.05$. For release of gene drive only, ${M}_{{WW},{WW}}={M}_{{WD},{WW}}=1/4$ and ${F}_{{WW},{WW}}={F}_{{WD},{WW}}=1/4.$ Assuming random mating, we obtain the following recursion equations for the female genotype frequencies in the next generation $(t+1)$:

$${F}_{{ij}{{\rm{|}}}{kl}}\left(t+1\right) \,=	 \frac{1}{2}\left(1-\frac{{\delta }_{{ij}}}{2}\right)\left(1-\frac{{\delta }_{{kl}}}{2}\right)\left({E}_{i{{\rm{|}}}k}\left(t\right){S}_{j{{\rm{|}}}k}\left(t\right)+ {E}_{j{{\rm{|}}}k}\left(t\right){S}_{i{{\rm{|}}}k}\left(t\right)\right.\\ \,	 +\, \left.{E}_{i{{\rm{|}}}l}\left(t\right){S}_{j{{\rm{|}}}l}\left(t\right)+{E}_{j{{\rm{|}}}l}\left(t\right){S}_{i{{\rm{|}}}l}\left(t\right)\right)$$

where ${\delta }_{{ij}}$ is the Kronecker delta. The factors $(1-\frac{{\delta }_{{ij}}}{2}),(1-\frac{{\delta }_{{kl}}}{2})$ account for the factor of 1/2 for homozygosity at the drive target site (for ${ij}=$ {*WW*, *DD*, *RR*}) and at the anti-drive site (for {${WW},{AA}$}). Similar equations may be written for the male genotype frequencies ${M}_{{ij|kl}}(t+1)$.

In the deterministic model, the load on the population incorporates reductions in female and male fertility and at time *t* is defined as:

$$L(t)=1-2F(t){\underline{w}}{\,\!}_{f}(t){\underline{w}}{\,\!}_{m}(t)$$

where ${\underline{w}}{\,\!}_{f}\left(t\right)={\sum }_{k=1}^{18}\;{{w}_{k}F}_{k}\left(t\right)/{\sum }_{k=1}^{18}\;{F}_{k}\left(t\right)\,$is the average female fitness and ${\underline{w}}{\,\!}_{m}\left(t\right)={\sum }_{k=1}^{18}\;{{w}_{k}M}_{k}\left(t\right)/{\sum }_{k=1}^{18}\;{M}_{k}\left(t\right)\,$is the average male fitness (here, *k* is summing over the 18 genotypes). $F\left(t\right)={\sum }_{k=1}^{18}\;{F}_{k}\left(t\right)$ is the proportion of females in the population ($=1/2$ except for the zeroth generation). The load is zero when only wild-types are present.

In the stochastic version of the model, as in refs. ^2^^,^^5^, probabilities of mating, egg production, hatching and emergence from pupae are estimated from experiments (Supplementary Table 2), and random numbers for these events are taken from the appropriate multinomial distributions. To model the cage experiments, 150 female and 30 male wild-type adults along with 120 male drive homozygotes (*WD*|*AA*), and 150 each female and male heterozygous for the *drive* (*WD*|*WW*) are initially present (600 individuals in total). For experiments with gene drive only and no anti-drive, there are 150 each of female and male *WD*|*WW* gene drive heterozygotes and 150 of wild-type adults. Females may fail to mate, or mate once in their life, with a male of a given genotype according to its frequency in the male population times its mating fitness (relative to wild-type), chosen randomly with replacement such that males may mate multiple times. The number of eggs from each mated female is multiplied by the egg production of the male relative to wild-type. To start the next generation, 650 eggs are randomly selected, and their hatching probability depends on the product of larval hatching values from the mother and father. The probability of subsequent survival to adulthood is assumed to be equal across genotypes. Assuming very large population sizes gives results for the genotype frequencies that are indistinguishable from the deterministic model. For the deterministic egg count, we use the large population limit of the stochastic model.

### Reporting summary

Further information on research design is available in the Nature Research Reporting Summary linked to this article.

## Supplementary information

Supplementary Information

Reporting Summary

## Data Availability

Raw-sequencing data were deposited in the EBI-ENA database under accession code PRJEB44729. The C77 plasmid sequence was deposited in the NCBI database under accession code MZ172909. Source data are provided with this paper.

## Source data

Source Data

## References

[1] Jinek m (2012). A programmable dual-RNA–guided DNA endonuclease in adaptive bacterial immunity. Science.

[2] Kyrou K (2018). A CRISPR–Cas9 gene drive targeting *doublesex* causes complete population suppression in caged *Anopheles gambiae* mosquitoes. Nat. Biotechnol..

[3] Hammond A (2016). A CRISPR-Cas9 gene drive system targeting female reproduction in the malaria mosquito vector *Anopheles gambiae*. Nat. Biotechnol..

[4] Gantz VM (2015). Highly efficient Cas9-mediated gene drive for population modification of the malaria vector mosquito *Anopheles stephensi*. Proc. Natl Acad. Sci. USA.

[5] Grunwald HA (2019). Super-Mendelian inheritance mediated by CRISPR–Cas9 in the female mouse germline. Nature.

[6] Simoni A (2014). Development of synthetic selfish elements based on modular nucleases in *Drosophila melanogaster*. Nucleic Acids Res..

[7] Gantz VM, Bier E (2015). Genome editing. The mutagenic chain reaction: a method for converting heterozygous to homozygous mutations. Science.

[8] Burt A (2003). Site-specific selfish genes as tools for the control and genetic engineering of natural populations. Proc. Biol. Sci..

[9] Windbichler N (2011). A synthetic homing endonuclease-based gene drive system in the human malaria mosquito. Nature.

[10] Hammond AM (2017). The creation and selection of mutations resistant to a gene drive over multiple generations in the malaria mosquito. PLoS Genet..

[11] Beaghton AK, Hammond A, Nolan T, Crisanti A, Burt A (2019). Gene drive for population genetic control: non-functional resistance and parental effects. Proc. R. Soc. B Biol. Sci..

[12] Unckless RL, Clark AG, Messer PW (2017). Evolution of resistance against CRISPR/Cas9 gene drive. Genetics.

[13] Champer J (2019). CRISPR gene drive efficiency and resistance rate is highly heritable with no common genetic loci of large effect. Genetics.

[14] Vella MR, Gunning CE, Lloyd AL, Gould F (2017). Evaluating strategies for reversing CRISPR-Cas9 gene drives. Sci. Rep..

[15] Esvelt KM, Smidler AL, Catteruccia F, Church GM (2014). Concerning RNA-guided gene drives for the alteration of wild populations. eLife.

[16] DiCarlo JE, Chavez A, Dietz SL, Esvelt KM, Church GM (2015). Safeguarding CRISPR-Cas9 gene drives in yeast. Nat. Biotechnol..

[17] Gantz VM, Bier E (2016). The dawn of active genetics. Bioessays.

[18] Wu B, Luo L, Gao XJ (2016). Cas9-triggered chain ablation of cas9 as a gene drive brake. Nat. Biotechnol..

[19] Xu X-RS (2020). Active genetic neutralizing elements for halting or deleting gene drives. Mol. Cell.

[20] Pawluk A (2016). Naturally occurring off-switches for CRISPR-Cas9. Cell.

[21] Rauch BJ (2017). Inhibition of CRISPR-Cas9 with bacteriophage proteins. Cell.

[22] Dong D (2017). Structural basis of CRISPR–SpyCas9 inhibition by an anti-CRISPR protein. Nature.

[23] Yang H, Patel DJ (2017). Inhibition mechanism of an anti-CRISPR suppressor AcrIIA4 targeting SpyCas9. Mol. Cell.

[24] Papathanos PA, Windbichler N, Menichelli M, Burt A, Crisanti A (2009). The *vasa* regulatory region mediates germline expression and maternal transmission of proteins in the malaria mosquito *Anopheles gambiae*: a versatile tool for genetic control strategies. BMC Mol. Biol..

[25] Simoni A (2020). A male-biased sex-distorter gene drive for the human malaria vector *Anopheles gambiae*. Nat. Biotechnol..

[26] Hammond A (2021). Regulating the expression of gene drives is key to increasing their invasive potential and the mitigation of resistance. PLoS Genet..

[27] Champer J (2018). Reducing resistance allele formation in CRISPR gene drive. Proc. Natl Acad. Sci. USA.

[28] Girardin L, Calvez V, Débarre F (2019). Catch me if you can: a spatial model for a brake-driven gene drive reversal. Bull Math Biol..

[29] Heffel MG, Finnigan GC (2019). Mathematical modeling of self-contained CRISPR gene drive reversal systems. Sci. Rep..

[30] Rode, N. O., Courtier-Orgogozo, V. & Débarre, F. Can a population targeted by a CRISPR-based homing gene drive be reduced? *G3 Genes Genom. Genet.* **10**, 3403–3415 (2020). 10.1534/g3.120.401484 PMC7466991 32727921

[31] Marshall R (2018). Rapid and scalable characterization of CRISPR technologies using an E. coli cell-free transcription-translation system. Mol. Cell.

[32] Mahendra C (2020). Broad-spectrum anti-CRISPR proteins facilitate horizontal gene transfer. Nat. Microbiol..

[33] Sitaraman K (2004). A novel cell-free protein synthesis system. J. Biotechnol..

[34] Fuchs S, Nolan T, Crisanti A (2013). Mosquito transgenic technologies to reduce *Plasmodium* transmission. Methods Mol. Biol..

[35] Pinello L (2016). Analyzing CRISPR genome-editing experiments with CRISPResso. Nat. Biotechnol..

