# Supplementary material for: A genetically encoded anti-CRISPR protein constrains gene drive spread and prevents population suppression
Source: Nat Commun. 2021 Jun 25;12:3977. doi: 10.1038/s41467-021-24214-5 (PMC8233359; doi:10.1038/s41467-021-24214-5)
Supplement: Supplementary file 1 — Supplementary Information [file 41467_2021_24214_MOESM1_ESM.pdf]

A genetically encoded anti-CRISPR protein constrains gene drive spread and prevents population suppression

Chrysanthi Taxiarchi, Andrea Beaghton, Nayomi Illansinhage Don, Kyros Kyrou, Matthew Gribble, Dammy Shittu, Scott P. Collins, Chase L. Beisel, Roberto Galizi & Andrea Crisanti

Supplementary Information

Supplementary Figures

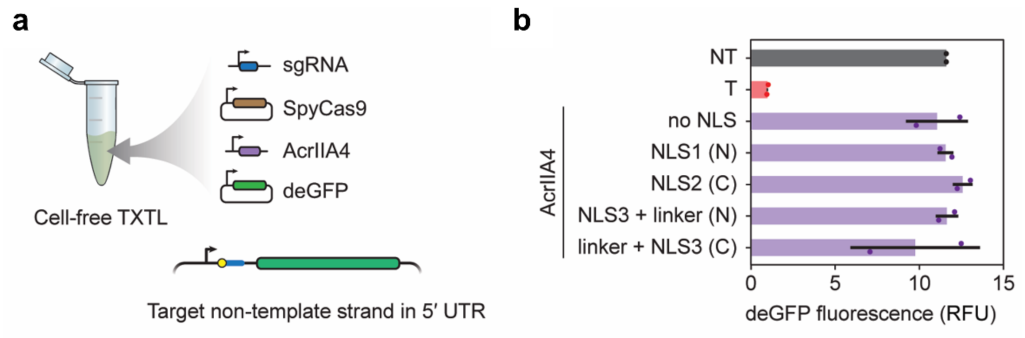

Supplementary Fig. 1. Inhibitory activity of AcrIIA4 unperturbed following the addition of NLS tags. (**a**) Assessing inhibition of SpyCas9 by AcrIIA4 using an *E. coli*-based cell-free transcription-translation system (TXTL). As part of the assay, SpyCas9 and an sgRNA targeting the deGFP construct are expressed, leading to cleavage and loss of deGFP expression. The presence of expressed AcrIIA4 inhibits DNA cleavage by SpyCas9, restoring deGFP expression. The components are encoded on linear DNA or on plasmids. (**b**) Assessing the impact of different NLS tags. Each tag was fused to the N-terminus (N) or C-terminus (C) of AcrIIA4. T: targeting sgRNA expressed without AcrIIA4. NT: non-targeting sgRNA expressed without AcrIIA4. NLS1 sequence: APKKKRKVGIHGVPAA. NLS2 sequence: KRPAATKKAGQAKKKK. NLS3 sequence: MPKKKRKV. Linker: SGGS. NLS sequences at the N-terminus begin with methionine to initiate translation. All NLS tags resulted in full restoration of deGFP expression. Values represent the mean and standard deviation of two independent measurements.

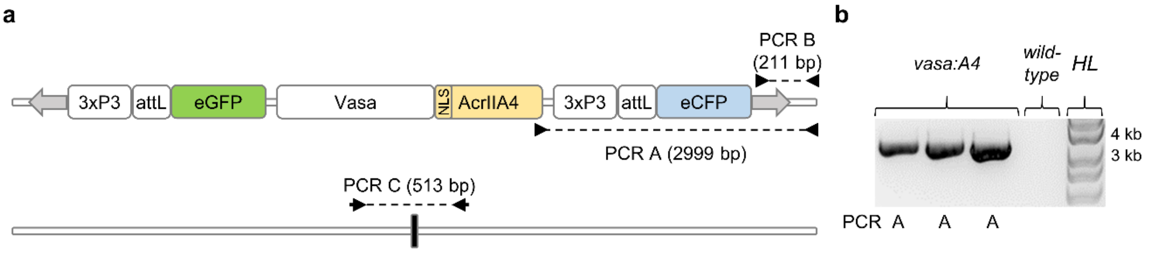

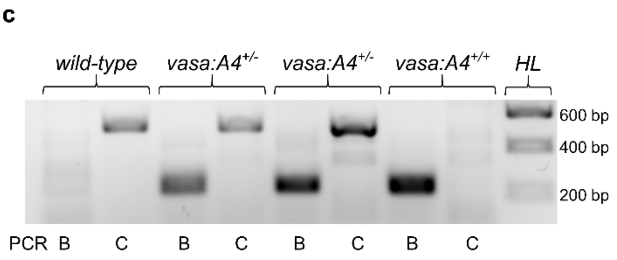

d

Genotype rates of vasa:A4 males released in the cage experiment		
**Genotype**	**N (%)**	**AV %**
vasa:A4$^{+/-}$	Cage 1: 5 (13.5 %)<br>Cage 2: 3 (12 %)	12.75 %
vasa:A4$^{+/+}$	Cage 1: 32 (86.5 %)<br>Cage 2: 22 (88 %)	87.25 %

Supplementary Fig. 2. Molecular characterization of the vasa:A4 transgenic line. (**a**) Schematic representation of the genomic integration of the vasa:A4 construct indicating the expected size of PCR fragments amplified using each set of primers (A, B and C). (**b**) Molecular confirmation of successful ϕC31 mediated integration of the vasa:A4 construct. Three biologically independent samples were tested giving positive confirmation of correct integration. One of these was randomly selected to establish the vasa:A4 transgenic line from the corresponding progeny. (**c**) Examples of PCR amplifications from genomic DNA extracted from single mosquitoes carrying one (*vasa:A4*$^{+/-}$) or two copies (*vasa:A4*$^{+/+}$) of the vasa:A4 construct and wild-type. A minimum of 25 individuals per cage were used for molecular confirmation of zygosity. (**d**) Proportion of heterozygous (*vasa:A4*$^{+/-}$) and homozygous (*vasa:A4*$^{+/+}$) anti-drive males released in the cage trial according to the PCR analysis shown in "C". HyperLadder1kb (HL) was used as molecular marker for DNA electrophoresis.

a

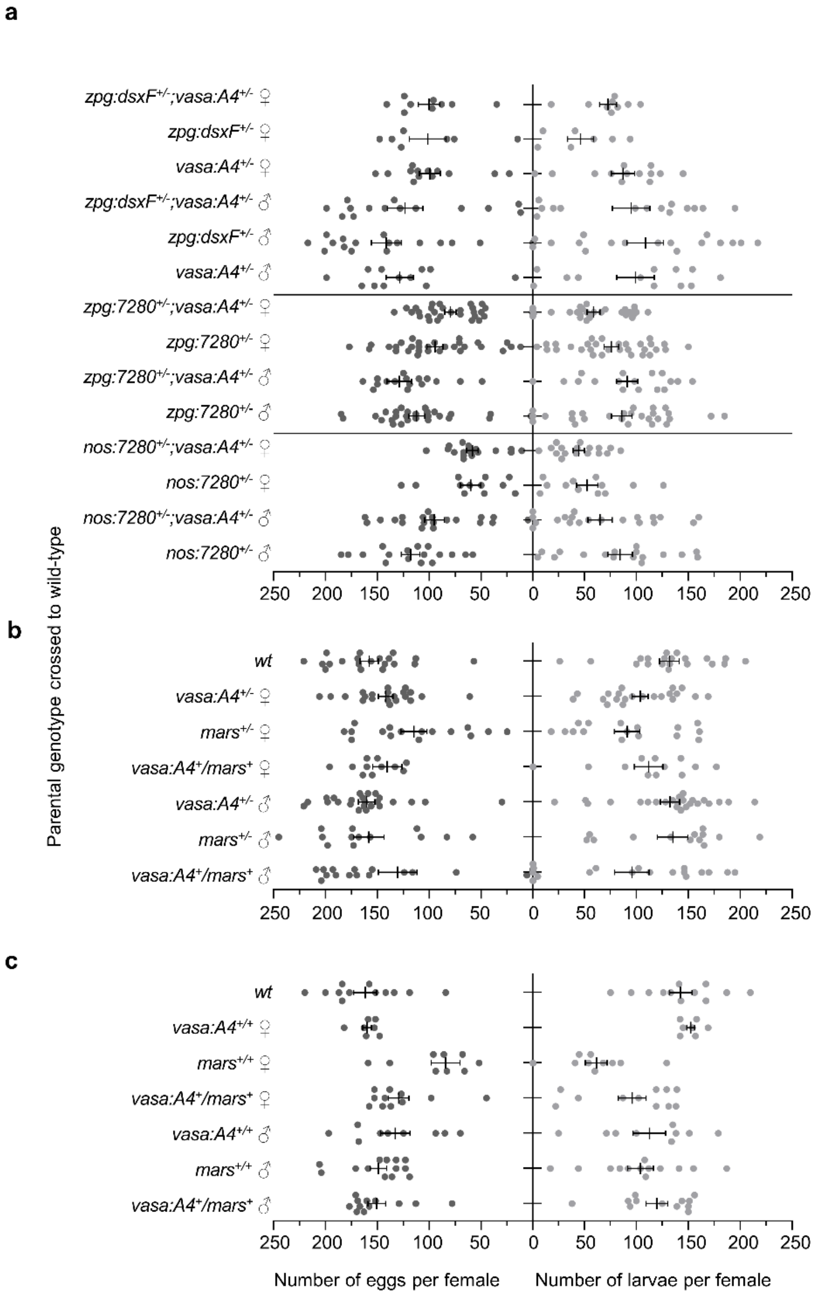

Eggs		Larvae		Total	
AV	NV	AV	NV	Eggs	Larvae
100.1	0.99	72.7	1.57	901	654**
101.4	R	46.1	R	710	323
99.5	0.98	87.2#	1.89	1293	1134**
123.2	0.87	94.9	0.87	1725	1329
141.4	R	108.6	R	2403	1846
128.3	0.91	99.1	0.91	1539	1189
79.6	0.84	58.8	0.77	2308	1704*
94.4	R	75.9	R	2831	2278
128.8	1.15	91.3	1.06	2190	1552
112.0	R	86.0	R	3025	2321
58.4	0.97	44.5	0.85	1051	756*
60.1	R	52.5	R	721	630
95.1	0.81	65.0	0.77	1902	1299
118.1	R	84.2	R	2007	1432

b

157.9	R	131.9	R	3157	2638
142.0	0.90	103.6#	0.79	2981	2175*
114.7#	0.73	91.2#	0.69	1835	1459
140.4	0.89	112.0	0.85	1685	1344
160.4	1.02	132.5	1.00	3850	3181
158.3	1.00	135.2	1.03	2058	1757
130.5	0.83	95.8	0.73	2349	1724*

c

161.8	R	142.7	R	1941	1712
159.7	0.99	152.4	1.07	1118	1067
84.2#	0.52	61.5##	0.43	842	615*
129.4#	0.80	96.1#	0.67	1423	1057*
132.8	0.82	112.6	0.79	1195	1013
149.0	0.92	103.9#	0.73	1937	1351**
150.6	0.93	119.8	0.84	1807	1438*

Supplementary Fig. 3. Fertility assays of gene drive and anti-drive transgenic lines. Scatter plots of the total number of eggs (dark grey dots) and larvae (light grey dots) counted from individual oviposition assays from wild-type mosquitoes crossed to transgenic females or males carrying: (**a**) one copy of the gene drive and/or anti-drive constructs; (**b**) one copy of the anti-drive constructs and/or one copy of a marker construct inserted at the same locus; (**c**) two copies of the anti-drive constructs or two copies of a marker construct inserted at the same locus (*vasa:A4/mars* crosses were also repeated for parallel reference). Error bars indicate mean values and standard error of the mean of number of eggs or larvae from all biological replicates assessed for each cross (also reported in the table on the **right** under average values (AV)). Normalised values (NV) were calculated against selected reference crosses (R) performed in parallel. Significance according to Welch's unpaired *t*-test (for both larval and egg output average values, indicated by "#") and Fisher's exact test (for the total number of hatched larvae, indicated by "*") was calculated against the reference cross ("*" or "#" corresponds to $P < 0.05$, ("**" or "##" corresponds to $P < 0.0001$). A minimum of 7

biologically independent samples (ovipositing females) were examined over one or two independent experiments for each cross. Raw data are provided in the Source Data file for Supplementary Fig. 3a, b and c.

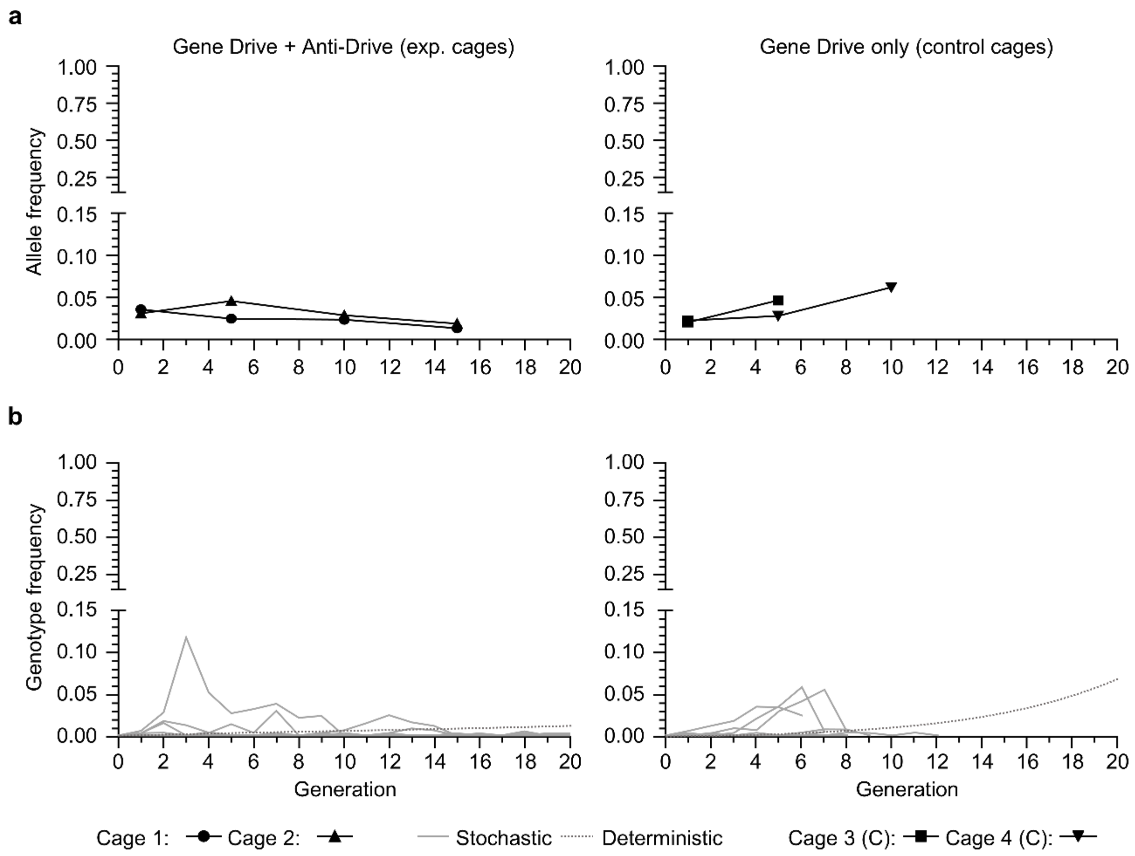

Supplementary Fig. 4. Resistance dynamics over generations at the *dsx*-target sequence. (**a**) Frequency plots of the total number of mutated alleles (indels and substitutions) among non-drive alleles, detected at the gRNA target sequence from generation 1, 5, 10 and 15 of the two experimental cages and generation 1, 5 and 10 of the two control cages. (**b**) Resistant genotype frequency trajectories modelled by deterministic (dotted line) or stochastic simulations (solid lines) over 20 generations (all the model parameters are equal to those used in **Fig. 3** and summarised in **Table S2**.

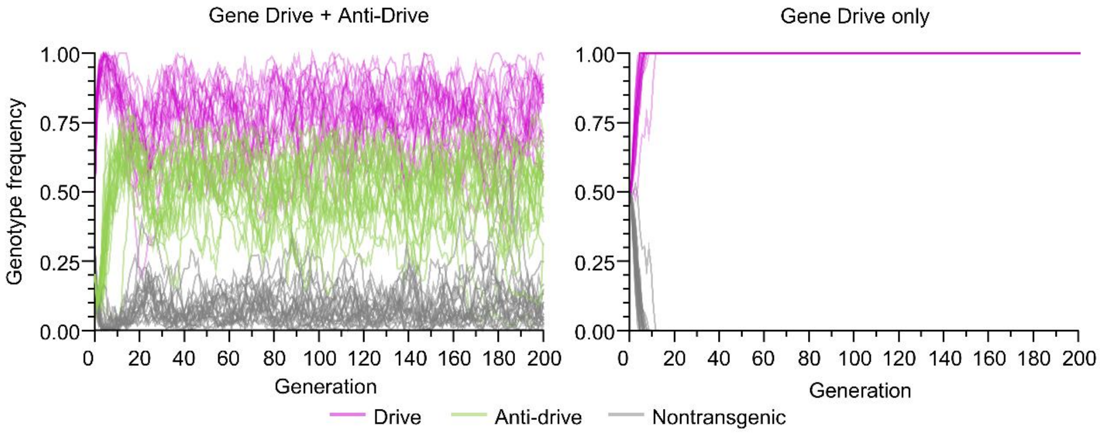

Supplementary Fig. 5. Stochastic dynamics of zpg:dsxF drive and AcrIIA4 anti-drive genotypes over extended time. Frequency over 200 generations of drive, anti-drive and nontransgenic individuals according to fitness parameters used for the cage trial models (**Fig. 3, and table S2**). The same starting frequencies were also applied, including the additional reduction in mating probability assumed for WW;AA males at G0 (0.2225 in G0 and 0.6 from G1 onwards).

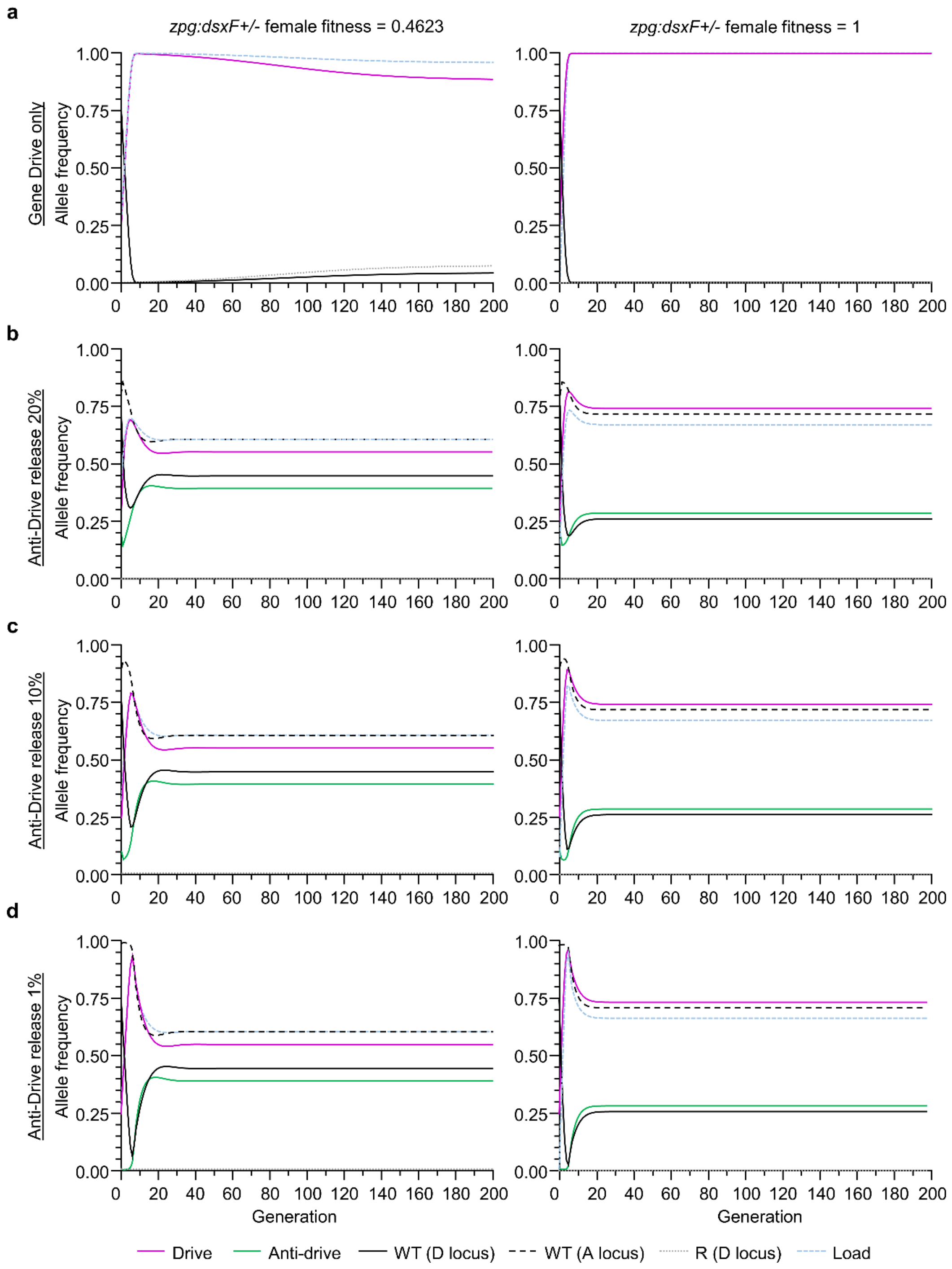

Supplementary Fig. 6. Effect of dive fitness on multigenerational dynamics of gene drive and anti-drive allele frequency. Deterministic model showing allele frequencies of drive, anti-drive, wild-type (WT, at both drive and anti-drive locus), non-functional resistant alleles (R, at the drive locus) and overall reproductive load assuming release of: (**a**) only 25% drive alleles for the control plots, (**b**) 25% drive and 20% anti-drive alleles, as used for the cage trial and stochastic models, (**c**) 25% drive and 10% anti-drive alleles or (**d**) 25% drive and 1% anti-drive alleles. Two different fitness values (relative to wild-type) of heterozygous gene drive females (WD;WW) were used: (**left**) equal to *zpg:dsxF*$^{+/-}$ females analysed in *Kyrou et al.* (0.4623), or (**right**) equal to wild-type (1.0).

Supplementary Tables S1-S3

**Parental genotype crossed to wild-type**	**Mating probability**		
	**Fraction**	**%**	***P***
wt	32/36	89	-
vasa:A4$^{+/-}$ ♀	21/26	81	0.4725
vasa:A4$^{+/-}$ ♂	24/30	80	0.4924
vasa:A4$^{+/+}$ ♀	7/10	70	0.1632
vasa:A4$^{+/+}$ ♂	9/15	60	0.0469

Supplementary Table 1. Mating probability of mosquitoes carrying one or two copies of the vasa:A4 construct. Fraction of mated females or males carrying one (*vasa:A4*$^{+/-}$) or two copies (*vasa:A4*$^{+/+}$) of the vasa:A4 construct scored in fertility assays. Fisher's exact (two-tailed) test was used to calculate significance against the wild-type control.

Drive locus	Anti-d. locus	Mating probability	Eggs per female	Hatching probability	Overall fitness	Meiotic EJ rate	Survival probability	Drive inheritance	Anti-drive inheritance
**Females**									
WW	WW	0.89 [1]	141.4 [3]	0.7680 [3]	1.0000	0	0.8708	0	0
WW	WA	0.81 [1]	99.5 [1]	0.8764 [1]	0.7308	0	0.8708	0	0.5
WW	AA	0.70 [1]	99.5 [6]	0.8764 [6]	0.6316	0	0.8708	0	1
WD	WW	0.89 [4]	76.1 [2]	0.4169 [2]	0.2921	0.4685	0.8708	0.999	0
WD	WA	0.81 [4]	75.2 [2]	0.6659 [2]	0.4197	0	0.8708	0.5	0.5
WD	AA	0.70 [4]	75.2 [6]	0.6659 [6]	0.3627	0	0.8708	0.5	1
WR	WW	0.89 [5]	141.4 [5]	0.7680 [5]	1.0000	0	0.8708	0	0
WR	WA	0.81 [5]	99.5 [5]	0.8764 [5]	0.7308	0	0.8708	0	0.5
WR	AA	0.70 [5]	99.5 [6]	0.8764 [6]	0.6316	0	0.8708	0	1
DD	WW	0.00	0.0	0.0000	0.0000	0	0.8708	1	0
DD	WA	0.00	0.0	0.0000	0.0000	0	0.8708	1	0.5
DD	AA	0.00	0.0	0.0000	0.0000	0	0.8708	1	1
DR	WW	0.00	0.0	0.0000	0.0000	0	0.8708	0.5	0
DR	WA	0.00	0.0	0.0000	0.0000	0	0.8708	0.5	0.5
DR	AA	0.00	0.0	0.0000	0.0000	0	0.8708	0.5	1
RR	WW	0.00	0.0	0.0000	0.0000	0	0.8708	0	0
RR	WA	0.00	0.0	0.0000	0.0000	0	0.8708	0	0.5
RR	AA	0.00	0.0	0.0000	0.0000	0	0.8708	0	1
**Males**									
WW	WW	0.89 [1]	141.4 [3]	0.7680 [3]	1.0000	0	0.8708	0	0
WW	WA	0.80 [1]	128.3 [1]	0.7724 [1]	0.8203	0	0.8708	0	0.5
WW	AA	0.60 [1,*]	128.3 [6]	0.7724 [6]	0.6152	0	0.8708	0	1
WD	WW	0.89 [4]	141.4 [1]	0.7680 [1]	1.0000	0.4685	0.8708	0.999	0
WD	WA	0.80 [4]	123.2 [1]	0.7703 [1]	0.7855	0	0.8708	0.5	0.5
WD	AA	0.60 [4]	123.2 [6]	0.7703 [6]	0.5891	0	0.8708	0.5	1
WR	WW	0.89 [5]	141.4 [5]	0.7680 [5]	1.0000	0	0.8708	0	0
WR	WA	0.80 [5]	128.3 [5]	0.7724 [5]	0.8203	0	0.8708	0	0.5
WR	AA	0.60 [5]	128.3 [6]	0.7724 [6]	0.6152	0	0.8708	0	1
DD	WW	0.89 [7]	141.4 [7]	0.7680 [7]	1.0000	0	0.8708	1	0
DD	WA	0.80 [7]	123.2 [7]	0.7703 [7]	0.7855	0	0.8708	1	0.5
DD	AA	0.60 [7]	123.2 [6]	0.7703 [6]	0.5891	0	0.8708	1	1
DR	WW	0.89 [7]	141.4 [7]	0.7680 [7]	1.0000	0	0.8708	0.5	0
DR	WA	0.80 [7]	123.2 [7]	0.7703 [7]	0.7855	0	0.8708	0.5	0.5
DR	AA	0.60 [7]	123.2 [6]	0.7703 [6]	0.5891	0	0.8708	0.5	1
RR	WW	0.89 [7]	141.4 [7]	0.7680 [7]	1.0000	0	0.8708	0	0
RR	WA	0.80 [7]	123.2 [7]	0.7703 [7]	0.7855	0	0.8708	0	0.5
RR	AA	0.60 [7]	123.2 [6]	0.7703 [6]	0.5891	0	0.8708	0	1

Supplementary Table 2. Parameters used for modeling. "W" indicates the wild-type allele at the drive (**left**) or anti-drive locus (**right**). "A" indicates the anti-drive allele. "D" indicates the drive allele. "R" indicates alleles causing non-functional resistance to the drive.[(1)] Average values obtained from phenotypic analysis performed in this work.[(2)] WD fertility values measured in this work were normalised for parental deposition in females measured in *Kyrou et al.* (maternal/paternal reduction rates: eggs per female = 0.50, hatching probability = 0.66).[(3)] Male fertility of WW;WW mosquitoes is considered equal to WD;WW males as in *Kyrou et al.*[(4)] Mating probability of WD individuals is considered equal to WW as in *Kyrou et al.*[(5)] Mating probability and fertility of WR males and females is considered equal to WW.[(6)] Fertility of AA mosquitoes is considered equal to WA. [(7)] Mating probability and fertility of DD, DR and RR males is considered equal to WD males (equal to WW) as in *Kyrou et al.* [(*)] An additional reduction in mating probability was assumed for WW;AA males at G0 (0.2225) for the cage trial models (**Fig. 3, S4, S5 and S6**). Inheritance values were rounded to 0.5 or 1 according to average values obtained from phenotypic analysis performed in this work. A 0.999 (instead of 1) value was used for WD;WW individuals to allow for R generation (0.4685 according to *Hammond et al. 2016*). Survival probability was also considered equal to *Kyrou et al.*

Primer name	5' to 3' sequence
RG427	AACCTCGAGATGCCGAAGAAAAAGAGGAAGGTGAGCGGCGGTAGCAACATTAATGATCTCATACGGGA
RG428	CGCTTAATTAATCAATTCAACTCGGACTTCA
RG1044	ATCCGTCGATGCCTAACTCG
RG187	TCAGGGGTCTTCAAACTTTATT
5R1	TGACACTTACCGCATTGACA
RG1047	AAGATAAGGGCTTGCCTCGG
*4050-Illumina-F	TCGTCGGCAGCGTCAGATGTGTATAAGAGACAGACTTATCGGCATCAGTTGCG
*4050-Illumina-R	GTCTCGTGGGCTCGGAGATGTGTATAAGAGACAGGTGAATTCCGTCAGCCAGCA

Supplementary Table 3. List of primers used in this study. Cloning overhangs are underlined with a single line and NLS sequence with wavy line. * Primers used for amplicon sequencing (Illumina adaptors underlined with double line).
