# Supplementary material for: A genetically encoded anti-CRISPR protein constrains gene drive spread and prevents population suppression
Source: Nat Commun. 2021 Jun 25;12:3977. doi: 10.1038/s41467-021-24214-5 (PMC8233359; doi:10.1038/s41467-021-24214-5)
Supplement: Supplementary file 2 — Reporting Summary [file 41467_2021_24214_MOESM2_ESM.pdf]

nature research

Corresponding author(s): Roberto Galizi, Andrea Crisanti

Last updated by author(s): 26/5/21

Reporting Summary

Nature Research wishes to improve the reproducibility of the work that we publish. This form provides structure for consistency and transparency in reporting. For further information on Nature Research policies, see our Editorial Policies and the Editorial Policy Checklist.

Statistics

For all statistical analyses, confirm that the following items are present in the figure legend, table legend, main text, or Methods section.

n/a	Confirmed	
☐	☒	The exact sample size (*n*) for each experimental group/condition, given as a discrete number and unit of measurement
☐	☒	A statement on whether measurements were taken from distinct samples or whether the same sample was measured repeatedly
☐	☒	The statistical test(s) used AND whether they are one- or two-sided<br>*Only common tests should be described solely by name; describe more complex techniques in the Methods section.*
☒	☐	A description of all covariates tested
☐	☒	A description of any assumptions or corrections, such as tests of normality and adjustment for multiple comparisons
☐	☒	A full description of the statistical parameters including central tendency (e.g. means) or other basic estimates (e.g. regression coefficient) AND variation (e.g. standard deviation) or associated estimates of uncertainty (e.g. confidence intervals)
☐	☒	For null hypothesis testing, the test statistic (e.g. *F*, *t*, *r*) with confidence intervals, effect sizes, degrees of freedom and *P* value noted<br>*Give P values as exact values whenever suitable.*
☒	☐	For Bayesian analysis, information on the choice of priors and Markov chain Monte Carlo settings
☒	☐	For hierarchical and complex designs, identification of the appropriate level for tests and full reporting of outcomes
☒	☐	Estimates of effect sizes (e.g. Cohen's *d*, Pearson's *r*), indicating how they were calculated

Our web collection on statistics for biologists contains articles on many of the points above.

Software and code

Policy information about availability of computer code

Data collection	JMicroVision V1.27 was used to count eggs in the cage trial
Data analysis	GraphPad Prism 6.0 was used for statistical analysis, CRISPResso v1.0.8 was used to analyse results from amplicon sequencing, Wolfram Mathematica was used for modeling calculations

For manuscripts utilizing custom algorithms or software that are central to the research but not yet described in published literature, software must be made available to editors and reviewers. We strongly encourage code deposition in a community repository (e.g. GitHub). See the Nature Research guidelines for submitting code & software for further information.

Data

Policy information about availability of data

All manuscripts must include a data availability statement. This statement should provide the following information, where applicable:

- Accession codes, unique identifiers, or web links for publicly available datasets
- A list of figures that have associated raw data
- A description of any restrictions on data availability

Raw amplicon sequencing data are available at the EBI-ENA database under accession code PRJEB44729 (Sup. Fig. 4). The C77 plasmid sequence is available at the NCBI database under accession code MZ172909.

Field-specific reporting

Please select the one below that is the best fit for your research. If you are not sure, read the appropriate sections before making your selection.

☒ Life sciences ☐ Behavioural & social sciences ☐ Ecological, evolutionary & environmental sciences

For a reference copy of the document with all sections, see nature.com/documents/nr-reporting-summary-flat.pdf

Life sciences study design

All studies must disclose on these points even when the disclosure is negative.

Sample size	Consistent with previous literature reporting similar phenotype assays (e.g., https://doi.org/10.1038/nbt.4245) the full progeny obtained from each oviposition/sample was counted/screened in each reported phenotype assay. Starting frequency, number of replicates and sampling used in th cage trial was consistent with previous literature reporting similar experiments (e.g., https://doi.org/10.1038/nbt.4245)
Data exclusions	No data exclusions
Replication	Biological replicates were performed as described in the main text. Consistent with previous literature (e.g., https://doi.org/10.1038/nbt.4245) a minimum of seven biologically independent samples (ovipositing females) were examined over 2 independent experiments for each cross/experiment.
Randomization	For the cage trial randomization was applied when selecting eggs to seed consecutive generations according to previous literature (e.g., https://doi.org/10.1038/nbt.4245)
Blinding	Blinding was not relevant to this study because none of the data recorded were subjective (i.e., number of larvae, eggs or mosquitoes positive for specific/unequivocal fluorescent markers).

Reporting for specific materials, systems and methods

We require information from authors about some types of materials, experimental systems and methods used in many studies. Here, indicate whether each material, system or method listed is relevant to your study. If you are not sure if a list item applies to your research, read the appropriate section before selecting a response.

Materials & experimental systems

n/a	Involved in the study	
☒	☐	Antibodies
☒	☐	Eukaryotic cell lines
☒	☐	Palaeontology and archaeology
☐	☒	Animals and other organisms
☒	☐	Human research participants
☒	☐	Clinical data
☒	☐	Dual use research of concern

Methods

n/a	Involved in the study	
☒	☐	ChIP-seq
☒	☐	Flow cytometry
☒	☐	MRI-based neuroimaging

Animals and other organisms

Policy information about studies involving animals; ARRIVE guidelines recommended for reporting animal research

Laboratory animals	The Anopheles gambiae G3 wild-type stock used in this study, originally procured from BEI Resources (MRA-112), has been in culture for over 30 years. Housing and experimental conditions followed standard procedures as described in the manuscript. For the purpose of giving mosquitoes a bloodmeal anesthetized female mice, age 2-6 months (CD1 strain) were used.
Wild animals	The study did not involve wild animals.
Field-collected samples	The study did not involve samples collected from the field.
Ethics oversight	All animal work was conducted according to UK Home Office Regulations and approved under Home Office License PPL 70/8914 by the AWERB at imperial College London.

Note that full information on the approval of the study protocol must also be provided in the manuscript.
